# Summary of current knowledge of the size and spatial distribution of the horse population within Great Britain

**DOI:** 10.1186/1746-6148-8-43

**Published:** 2012-04-04

**Authors:** Lisa A Boden, Tim DH Parkin, Julia Yates, Dominic Mellor, Rowland R Kao

**Affiliations:** 1 Boyd Orr Centre for Population and Ecosystem Health, Institute of Biodiversity, Animal Health and Comparative Medicine, College of Medical, Veterinary & Life Sciences, University of Glasgow, 464 Bearsden Road, Glasgow G61 1QH, UK; 2 Boyd Orr Centre for Population and Ecosystem Health, School of Veterinary Medicine, College of Medical, Veterinary and Life Sciences, University of Glasgow, 464 Bearsden Road, Glasgow G61 1QH, UK

**Keywords:** Equine, Demography, Spatial distribution, Infectious disease, The National Equine Database

## Abstract

**Background:**

Robust demographic information is important to understanding the risk of introduction and spread of exotic diseases as well as the development of effective disease control strategies, but is often based on datasets collected for other purposes. Thus, it is important to validate, or at least cross-reference these datasets to other sources to assess whether they are being used appropriately. The aim of this study was to use horse location data collected from different contributing industry sectors ("Stakeholder horse data") to calibrate the spatial distribution of horses as indicated by owner locations registered in the National Equine Database (the NED).

**Results:**

A conservative estimate for the accurately geo-located NED horse population within GB is approximately 840,000 horses. This is likely to be an underestimate because of the exclusion of horses due to age or location criteria. In both datasets, horse density was higher in England and Wales than in Scotland. The high density of horses located in urban areas as indicated in the NED is consistent with previous reports indicating that owner location cannot always be viewed as a direct substitute for horse location. Otherwise, at a regional resolution, there are few differences between the datasets. There are inevitable biases in the stakeholder data, and leisure horses that are unaffiliated to major stakeholders are not included in these data. Despite this, the similarity in distributions of these datasets is re-assuring, suggesting that there are few regional biases in the NED.

**Conclusions:**

Our analyses suggest that stakeholder data could be used to monitor possible changes in horse demographics. Given such changes in horse demographics and the advantages of stakeholder data (which include annual updates and accurate horse location), it may be appropriate to use these data for future disease modelling in conjunction with, if not in place of the NED.

## Background

Understanding how an infectious disease might spread through a population and how then to control that spread requires knowing both the size of the susceptible population at risk and, and how frequently individuals in a population come into contact with one another. In the case of disease spread at a national scale, this requires knowledge of the spatial distribution of the susceptible population, as this will inform 'local' spread that transmits simply as a result of geographical proximity, and can be used to parameterise 'network-based' spread, such as can occur via livestock movements. Great Britain (GB) needs to prepare for a potential equine infectious disease epidemic due to the recent incursion of Bluetongue virus (BTV) from North Africa through Europe, and the increase in the number of African Horse Sickness Virus (AHSV) serotypes within the historical northern limits of the virus' range in sub-Saharan Africa [1]. Once an incursion of AHSV occurs in Europe (or the Middle East), where there are major centres of international movement of horses, the consequences could be economically devastating [1]. Thus, the availability of robust demographic information is important to understanding the risk of introduction and potential spread of an outbreak of this and other exotic equine diseases (should one occur) as well as for the development of preventive disease control strategies. In the event of an infectious disease outbreak, decisions may be necessarily based on models which are parameterised from data recorded for purposes other than epidemiological demographic studies. Therefore, it is necessary to validate such databases to ensure that they are being used appropriately and to estimate the degree of uncertainty associated with outcomes from these models.

While demographic data for British livestock are generally available, demographic data for the equine population within GB are poor. A number of surveys have been conducted in the UK to estimate demographic attributes of the equine population [2-6]. The results of these surveys vary with respect to estimates of horse numbers (between 600,000 and 1.3 million [7-10]) and should be interpreted cautiously because of the diverse nature of data collected by different authorities (UK, GB, England, Scotland, Wales) and small numbers of survey respondents [11], to say nothing of the datedness of some of these estimates. Since 2006, the National Equine Database (NED) has received data on all equidae issued with a passport from any of the 80 passport issuing organisations (PIOs) in the UK. This includes information on horse identification and owner address. A horse passport has been mandatory for all owned horses since 2004. The current horse passport system relies on the horse owner to update passports (and owner addresses) when horses are bought, or have died. In 2010, there were an estimated 21,000 horse owners registered online with the NED. Of these, only 11% have associated themselves with their horse(s) (*pers. comm*. Doug Stephens, the NED). Owners need to subscribe to the NED and associate themselves with their horse in order to check that the details (such as home address) for their horse are correct. As such, the number and location of equidae in the NED may be inaccurate; inevitably there will be some inclusion of horses with multiple passports, fraudulent passports, horses that have foreign passports but reside in GB, horses that have GB passports but reside outside of GB and dead horses (whose owners have not returned their passports). In addition, horses may well not reside at the same location as the owner's address [7,12] and some horses may not have a passport [7]. As the NED has been only recently implemented, these potential inaccuracies in the data may be small in number and thus may not seriously compromise the quality of the data for use in disease control models [13]. However, as yet, the extent of these errors in the NED have not been formally assessed or documented.

The aim of this study was to examine the data quality of the NED and critically examine the discrepancies between the spatial distributions of horses in the NED and independently collected equine demographic data provided by other stakeholders in the equestrian industry (at regional and postcode area resolutions).

## Methods

### Data collection

In this study, the term "horses" refers to horses, ponies, donkeys, zebras or any animal produced by crossing these species.

A list of the contact details of all passport issuing organizations was obtained from the NED. A list of contact details for other equine organizations was obtained online from the British Equine Federation (http://www.bef.co.uk/About_the_BEF/Member_Bodies.html). Weatherbys (racing division and stud book) was contacted for data on Thoroughbred horses used for racing and breeding. Equine welfare charities (such as World Horse Welfare, Redwings, British Horse Society, Donkey Sanctuary etc.) were identified from an online search using the terms 'equine', 'horse', 'charity', 'welfare' and 'Great Britain'. All equestrian organisations and stakeholder groups were contacted by telephone and email to obtain data on horse location and movements. A consultation list of industry members is provided in Additional file 1: Table S1. Data were also obtained on horse and owner location held by Defra. This included Agricultural Census data (horse location) and summary data from the Scotland Government Census [14] (horse location) and the National Equine Database (NED) (horse owner location).

### Data analyses

Data which contained horse rather than owner or member location (e.g. from the agricultural census, BHA, Weatherbys, British Eventing, World Horse Welfare, Redwings and the Donkey Sanctuary) were aggregated to create a Stakeholder horse location dataset. Data from the NED, which represents owner location for all recorded horses is henceforward referred to as the NED owner dataset.

All data supplied in the NED owner dataset and in the Stakeholder horse dataset were anonymous with respect to horse name and/or owner/dealer name and specific address. Data were summarised to represent the number of horses in 121 postcode areas in mainland GB. Postcode areas are represented by the initial alphanumeric characters in the postcode. These are intended as a mnemonic for the places served (http://www.postcode-info.co.uk/postcode-areas.html). Postcodes for Northern Ireland and the Crown dependencies (Isles of Jersey, Guernsey and Man) were excluded from these analyses to restrict the study to postcode areas within 'mainland' GB. Postcode areas were classified into eleven regions (East England (E), Greater London (GL), East Midlands (EM), West Midlands (WM), North East (NE), North West (NW), Yorkshire and Humber (YH), South East (SE), South West (SW), Wales and Scotland). Greater London was ascribed as a separate region so that specific comparisons could be made between the datasets. The numbers of horses and percentages of all horses in each postcode area and region were calculated from the NED owner dataset and from the Stakeholder horse dataset.

The NED owner dataset was further restricted by age to examine the impact of exclusion of implausibly old horses. Given that the NED database is reasonably new, these old horses may have arisen due to incorrectly entered birthdates or from historical records inherited from PIOs which were never removed on the event of a horse's death. The birth dates of horses registered in the NED were obtained if they were recorded and the current age (in 2010) of horses calculated. The spatial distribution of number (and percentage) of horses per postcode area was examined using different horse age cutpoints (≤ 30, ≤ 40, ≤ 50 and ≤ 60 years). These restricted datasets were compared with the original complete NED data using Bland Altman limits of agreement plots to identify postcode areas where there were extreme numbers of older or younger horses. Bland-Altman plots are used to determine the level of agreement between two different measures (in this case, measures of horse location). The difference between two measurements against the mean of the two measurements is plotted, thereby allowing investigation of any possible relationship between the measurement error and best estimate of the true value (i.e. the mean of the two measurements) [15]. Ultimately, the NED owner dataset was restricted to horses which had valid postcode areas within their addresses and were 30 years of age or younger. Previous work on geriatric horses estimates that the median age for geriatric horses is 20 years (with only 5% of the geriatric population aged greater than 30 years) [16]. A previous study [6] estimated that horses in northern Britain ranged in age from 1 month to 37 years (mean 11 years).

Neither dataset (Stakeholder horse or the NED owner dataset) was considered to be a 'gold standard' measure of the horse population in GB, and therefore it was not considered appropriate to use either dataset to estimate the total horse population accurately. In order to calibrate the spatial distribution of the NED owner dataset, the total number of horses (still likely to be alive) in the NED was taken as the best estimate of total horse population size. The number of horses in the Stakeholder horse dataset was normalised by calculating the proportional distribution of horses per postcode area. This distribution of the number of horses in the horse dataset was then scaled upwards to reflect the equivalent total size of the horse population represented in the NED owner data. On this basis, horse densities (per 10 km^2^) for each postcode area and geographical region within mainland GB were calculated and mapped for both the NED owner and horse datasets.

### Comparison of horse location data with age-restricted data from the NED

The spatial distributions of the NED owner and horse datasets were compared graphically and using maps (created in R statistical software). Bland-Altman limits of agreement plots were used to identify important differences in the number of horses within postcode areas in each dataset [15]. In this study, these plots were used to calculate the limits within which the two datasets (the NED owner and Stakeholder horse datasets) can be considered equivalent. These plots were used to estimate the uncertainty around using the mean of the two datasets as an estimate of horse numbers per postcode area and per region.

## Results

### Stakeholder horse dataset

The Stakeholder horse dataset included valid geographical postcode and region locations for 35,841 horses registered for competition related pursuits (i.e. 16,010 Thoroughbred horses in race training (BHA); 10,055 Thoroughbreds used for breeding (Weatherbys) and 9,776 horses registered for British Eventing). Data on owner location were also routinely recorded by British Dressage and Endurance GB and many specific breed associations. However, these were not incorporated in further analyses as they did not specifically identify horse as opposed to owner or member location. Horse location data were also available on 5,613 registered and unregistered horses assigned to welfare associations (i.e. associated with the Donkey Sanctuary (n = 3,269), World Horse Welfare (n = 1,890), and Redwings Horse Sanctuary (n = 454)). Additionally, data on horse locations were available for all registered and unregistered horses kept on British farms, as recorded in the Agricultural Census (n = 358,231 horses on 58,492 holdings). Of these, 346,211 horses were ascribed to a valid mainland GB postcode.

These data were a convenience sample and thus were not considered to be representative of the true spatial distribution of the general equine population. Stakeholder horse data were subsequently aggregated to describe a summary measure of the spatial distribution of horses within mainland GB. Due to the summary nature of the data collected for each of these sectors, it is possible that there could be overlap and possible double counting of horses amongst some of the datasets. However, it was felt that this was unlikely to have a great impact on results for the horse dataset due to the reasonably small numbers of horses in each sector outwith the agricultural census data (n = 41,454 non-agricultural horses; 11% of Stakeholder horse data).

### The NED owner data

According to the NED, slightly more than half the horses within mainland GB are registered horses (57%) (i.e. horses registered on a studbook of a registered breed society (33%) or holding a passport from an organisation that holds international competitions (e.g. British Equestrian Federation, Weatherbys) (24%)).

In June 2010, there were 1,383,304 horses with passports recorded in the NED. Of these, there were 308,583 (22%) horses that had to be excluded from further consideration in this study. Of these exclusions, 4,867 horses (1.6%) were recorded in the NED as 'no recorded address', 65,994 horses (21.4%) as 'no fixed abode', 237,722 horses (77%) had addresses recorded but no valid matching postal code. Of the 237,722 horses which had an address, but no valid postcode, 162,131 (68%) horses could not be ascribed any address within or outwith the UK. The remaining horses (75,591 or 32%) had foreign passports and/or foreign addresses. A further 39,814 horses not resident on mainland GB were excluded: Northern Ireland (95% of 39,814 horses), Isle of Man (2.6%), Jersey (1.3%) and Guernsey (1.1%). Ultimately there were 1,034,907 horses that had owners with valid postcode areas and which could be included in further demographic analyses.

Birthdates were obtained for 903,805 horses in the NED owner dataset. Horses born before 1950 (n = 240) and horses recorded as 'born' after 2010 (n = 48) were excluded. The median age was 16.5 years (mean age 12.8 years, range < 1 year to 60 years) for all horses born between 1950 and 2010 (n = 903,517). The majority of horses within this age range (93%, n = 842,653) were aged less than 30 years. When the spatial distribution of horses aged 30 years or younger was compared with that of the full NED owner dataset, there were more old horses (30 years or older) than expected in the Norwich (NR) and Exeter (EX) postcode areas. There were more horses less than 30 years old than expected in Cardiff (CF), Southampton (SO) and Swansea (SA) postcode areas.

After all of these exclusions described above, were made, a low estimate for the population of horses in mainland GB which are most likely to be alive (30 years old or less) and have valid postcodes is 842,653 horses.

**Figure 1:**
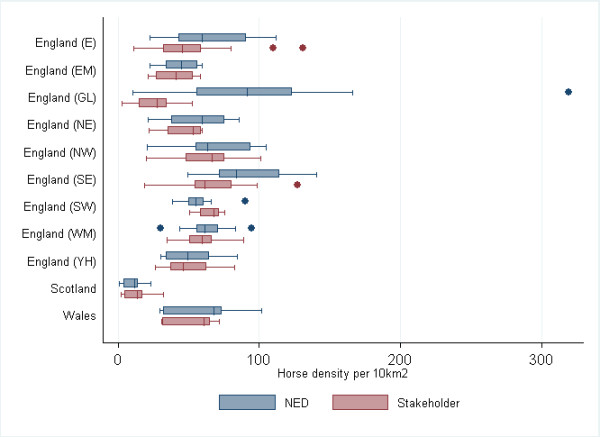
**Comparison between the NED owner and Stakeholder horse data compiled from other sources describing the distribution of the density (per 10 km^2^) of owners/horses within postcode areas within regions within Great Britain (East England (E), Greater London (GL), East Midlands (EM), West Midlands (WM), North East (NE), North West (NW), Yorkshire and Humber (YH), South East (SE), South West (SW), Wales and Scotland)**. The density of horses in London (GL) in the NED owner dataset is considerably higher than that in the Stakeholder horse dataset. Otherwise, at a regional level, NED owner and Stakeholder horse datasets appear to be very similar.

**Figure 2:**
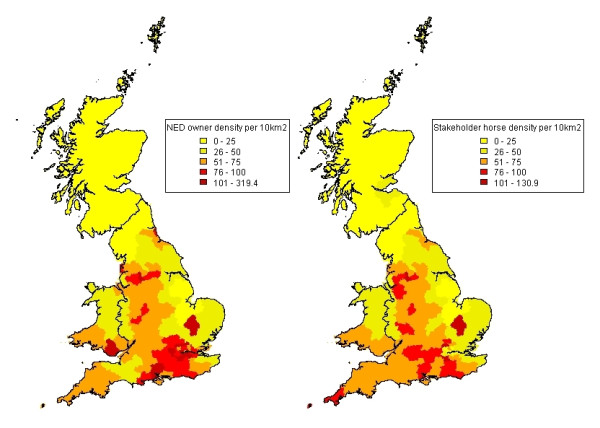
**Maps of the NED owner and Stakeholder horse data at postcode area resolution**. The legend represents horse density per 10 km^2^. Compared to the Stakeholder horse dataset, the NED owner dataset appears to have higher densities of horses in urban areas such as London. This supports previous views that owner location is not a good proxy measure for horse location in certain parts of the country. Apart from this, the distributions of horse location in the two datasets are very similar.

The distribution of horses per postcode area within each region within mainland GB is described for the NED owner and Stakeholder horse datasets in Figure 1. Maps of the density of horses per postcode area using the NED owner and Stakeholder horse datasets are illustrated in Figure 2. The majority of horses are concentrated in England (82% in both datasets), with a smaller percentage of horses residing in Wales (11% the NED owner dataset, 9% Stakeholder horse dataset) and Scotland (7% the NED owner dataset, 8% Stakeholder horse dataset). Estimated horse density was greater in England (51 horses per 10 km^2 ^in both datasets) and Wales (54 per 10 km^2 ^in the NED owner dataset; 48 horses per 10 km^2 ^in Stakeholder horse dataset) than in Scotland (7 horses per 10 km^2 ^in the NED owner dataset, 9 horses per 10 km^2 ^in Stakeholder horse dataset). The NED ascribes a London address to 7,432 horses (0.88% of all horses with a valid postcode area) whereas the Stakeholder horse dataset ascribes a London address to 1,749 horses (0.21% of all horses with a valid postcode area). In the NED owner data, London had the greatest density of horses (104 horses per 10 km^2^), whereas London had the lowest density of horses in England in the Stakeholder horse dataset (25 horses per 10 km^2^). In Wales, Cardiff had the greatest density of horses reported in both datasets, although this was higher in the NED owner dataset compared to that of the Stakeholder horse dataset (102 horses per 10 km2 and 72 horses per10 km^2^, respectively). In Scotland, the greatest density of horses was reported in the Kircaldy postcode area in both datasets (23 per 10 km^2 ^in the NED owner dataset; 32 horses per 10 km^2 ^in Stakeholder horse dataset).

**Figure 3:**
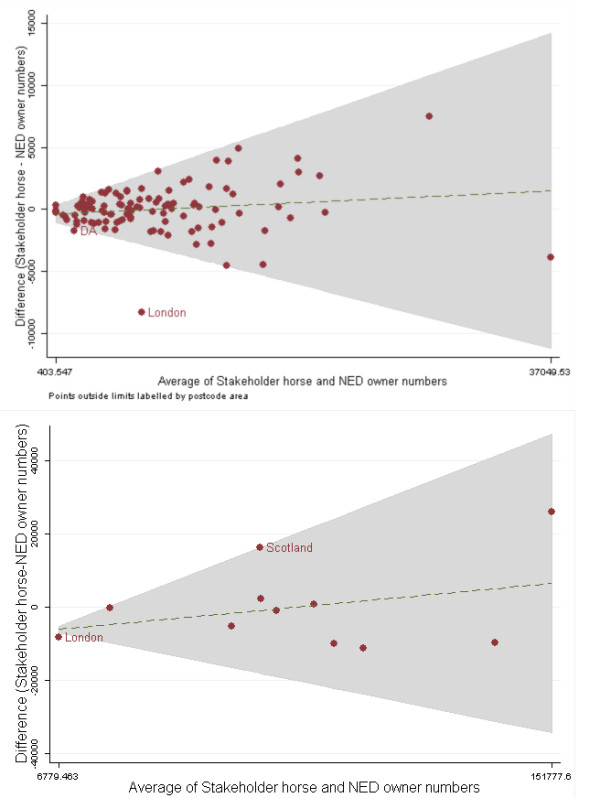
**Bland-Altman plots showing the limits of agreement between the numbers of horses per postcode area and by region in the NED owner and Stakeholder horse datasets**. These plots assume a relationship between the mean number of horses and the difference in the number of horses in each postcode area or region. The plots illustrate the agreement between the relative percentages of horses in each postcode area/region in the Stakeholder horse and the NED owner data. The owner location data provided by the NED has been restricted to include only those horses less than 30 years old. There are comparatively fewer horses in the Stakeholder horse dataset compared to the NED owner dataset in Dartford (DA) and London. Scotland appears to have comparatively greater numbers of horses in the Stakeholder horse dataset compared to the NED owner dataset.

Bland-Altman limits of agreement plots illustrating the uncertainty around the mean number of horses in the two datasets per postcode area and per region are described in Figure 3. There were comparatively fewer horses in the Stakeholder horse dataset in the Dartford (DA) and London postcode areas. At a regional resolution, the distribution of numbers of horses in England and Wales was similar. In addition to the difference in horse numbers in London, the most significant difference between the two datasets was the greater number of horses in Scotland in the Stakeholder horse dataset compared to the NED owner dataset.

## Discussion

This study presents our best estimate of the spatial distribution of horses which can be geo-located within mainland GB from the NED (n = 842,653 horses) at both regional and postcode area resolutions. The number of horses represented in this study is likely to be an underestimate of the true size of the horse population. A further approximately 500,000 horses may reside in the UK, but it is not clear from the NED that they are still alive or where they are located. In addition, it is not currently possible to produce an estimate for the numbers of horses which are registered within mainland GB, but which may have been subsequently exported to EU or other foreign countries. The origin of horses which have re-registered with a UK passport is also untraceable under the current system. The likely maximum estimate of the horse population in mainland GB is therefore in the region of 1,350,000 horses.

Apart from Scotland, the spatial distribution of horses, at the regional level, in the NED owner dataset was not very different to that of the Stakeholder horse data. However, important differences were apparent at the level of the postcode area. Compared to the Stakeholder horse dataset, there was an ostensibly higher density of horses in London in the NED owner dataset. This supports the previously held view that owner location cannot be viewed as a direct substitute for horse location in certain parts of the country [7]. It also suggests that models parameterized with the NED owner data may incorrectly overestimate the impact of disease introduction and spread in these areas relative to others. However, it is also important to note that the number of horses reported to reside in these urban locations is small (approximately 1% of the total equine population of mainland GB) and as such the impact of these inaccuracies may be minimal in terms of GB-wide disease modeling or spread. The differences between datasets are attributable in part to the inevitable bias in the Stakeholder horse location dataset and in part to inaccuracies in the NED and could have an impact on future studies which attempt to model disease spread at different geographical resolutions.

Reassuringly, differences between datasets were minimal at a regional level, but at higher data resolutions (postcode areas) meaningful and potentially concerning differences were detected. This would suggest that these data could be used, for example, to parameterise the relative likelihood of contact (e.g. via horse movements) between geographic regions, and the relative likelihood that disease would persist in those regions, if introduced. However, this observation puts important limits on how fine the resolution at which detailed model predictions and recommendations can be confidently made to aid disease control. The importance of high resolution horse demography data depends on the type of pathogen involved in an infectious disease outbreak and disease control policy implemented. On one hand, regional data may be sufficient (for example in vector-borne disease models), but perhaps when dealing with non-vector-borne diseases, postcode area or still finer resolution data may be more desirable. Whilst the precise effects of the differences between datasets at different resolutions are difficult to predict, the risk exists that decisions based on more generic analyses at a lower resolution are likely to be less effective at a local level. Over-interpretation of any dataset, particularly when used for predictive modeling, may lead to errors being made in terms of disease control methods applied to individual animals in the face of a disease outbreak. These decisions may not only result in regrettable consequences for animals and their owners, but could well also erode public confidence in and compliance with, scientific advice.

Obtaining independently collected data on horse location from sectors of the industry (Stakeholder horse data) was difficult due to the diverse and fragmented nature of the 'equestrian industry' within GB. Apart from the data collected in the Stakeholder horse dataset, there is no other centralised database which is maintained by the equestrian industry which could be used independently to cross-reference the data in the NED. Although other important sectors of the equestrian industry (apart from the affiliations mentioned in the Stakeholder horse dataset) collect demographic data, these are typically recorded as owner rather than horse location. As expected, horse location data are routinely and rigorously collected for many registered horses (such as Thoroughbreds in racing and breeding and other competitions such as eventing). However, these sectors of the industry represent a very small proportion of all horses within GB. As a result, the spatial distribution of the horses in the horse dataset is largely driven by the location of horses on agricultural land (41% of the Stakeholder horse data). Clearly, both the NED owner and the integrated stakeholder datasets have their limitations. It is likely that horses not currently included in the Stakeholder horse dataset, will follow a different spatial distribution than reported here. This is an obvious bias associated with such Stakeholder data provided by individual sectors of the equine industry. The majority of horses not included in the Stakeholder dataset are likely to be classified as leisure horses used for riding and other leisure purposes and it is possible they are kept within livery yards or stables and have a more clustered distribution within urban and semi-urban areas.

In real time, the location of many horses in GB is likely to be far more dynamic than can be captured by any database. Even if the horse registration location is known and it is considered plausible that this is where the horse is kept, it does not necessarily represent where a horse is located on any given day or even from month to month or year to year. For example, many mares will leave their residence to go to stud for breeding in the spring. However, in the event of a disease outbreak in the GB horse population, national or regional decisions may have to be made regardless of whether or not detailed, validated population information is available. It is therefore reassuring that the two relatively independent estimates of population distribution (the NED owner and Stakeholder horse datasets) vary so little in the vast majority of mainland GB, though results from disease models based on any (or combinations) of these datasets would need to be interpreted cautiously in light of the uncertainty which exists between real-time horse location compared to what is recorded as horse or owner residence. Neither the NED dataset nor the aggregated stakeholder data were designed to aid disease control. Although the creation and maintenance of a single database that collates the horse population in its entirety has considerable value, it is critical that such data be validated against independent sources.

## Conclusions

Knowledge of the spatial distribution of the horse population is important both for determining whether a region is likely to be exposed to an infectious disease and also whether it would persist once introduced. Previously there has been no assessment of data quality within the NED, which is the only broadly collected source of data on the GB horse population. The independently acquired Stakeholder horse data show a remarkably similar distribution to the NED dataset, implying that they are a useful adjunct, providing an independent source of horse demography data alongside the NED. The Stakeholder horse data are recorded annually and thus provide a dynamic representation of the horse population. Our analyses also suggest that the Stakeholder horse data could be used to monitor possible changes in horse demographics. Given such changes in horse demographics and the advantages of stakeholder data (annual updates and accurate horse location) it may be appropriate to use these data for future disease modelling in conjunction with, if not in place of the NED.

## Competing interests

The authors declare that they have no competing interests.

## Authors' contributions

LB, JY, TP, DM, RK participated in the design of the study. LB and JY collected data and performed the statistical analyses. LB, TP, DM, RK conceived of the study, and participated in its design and coordination and helped to draft the manuscript. All authors read and approved the final manuscript.

## Supplementary Material

Additional file 1 **Table S1 Consultation list of members of the equestrian industry in GB**. Click here for file
