# Supplementary material for: Summary of current knowledge of the size and spatial distribution of the horse population within Great Britain
Source: BMC Vet Res. 2012 Apr 4;8:43. doi: 10.1186/1746-6148-8-43 (PMC3351363; doi:10.1186/1746-6148-8-43)
Supplement: Additional file 1 — Table S1 Consultation list of members of the equestrian industry in GB. [file 1746-6148-8-43-S1.DOCX]

**Additional File 1:**

**Table 1. Consultation list of members of the equestrian industry in GB.**

Agricultural census

The NED

British Show Jumping Association

American Saddlebred Association of Great Britain

BHA data

Horse Passport Agency Ltd, Fell Pony Society

Fjord Horse Studbook Society

Weatherbys

Standard and Trotting Horse

Association of Great Britain and Ireland

American Miniature Horse Club

British Dressage

Welsh Pony & Cob Society

British Appaloosa Society

British Carmargue Horse Society

Trekking centres

Pet-ID UK Ltd

Horse Passports Ltd

National Equine Database Ltd (EU non-UK Equines)

Saddlebreds

Weatherbys ID Passports

British Connemara Pony Society

Selle Francais EquiCours

Endurance Sport Horse Breeding of Great Britain

Scottish Sports Horse

British Equestrian Federation (EU non-UK Equines)

British EventingShetland Pony Stud-Book Society

Warmblood Breeders Studbook UK

Lipizzaner Society of Great Britain

Pony Club

Pleasure Horse Society

Cleveland Bay Horse Society

Show Jumping Association of Ireland Ulster Region

TRACES

British Horse Society

International Miniature Horse and Pony Society

Northern Ireland Horse Board Co-op Society Ltd

Zoo

Arab Horse Society

Shire Horse Society

British Show Jumping Association

Horse market data

New Forest Pony Breeding & Cattle Society

Exmoor Pony Society

Fell Pony Society

RDA

Veteran Horse Society

British Hanoverian Horse Society

Standard and Trotting Horse Association of Great Britain and Ireland

Donkey Sanctuary

British Driving Society

British Equestrian Federation

British Appaloosa Society

Donkey Breed Society

Trakehners UK

Horse Passports Ltd

Anglo-European Studbook Ltd.

Gypsy Cob Society

British Connemara Pony Society

Dartmoor Pony Society

Dales Pony Society

Scottish Sports Horse

Hurlingham Polo Association

American Quarter Horse Association UK

Warmblood Breeders Studbook – UK

National Pony Society

Spotted Horse and Pony Society

Cleveland Bay Horse Society

Irish Draught Horse Society(GB)

British Association for the Purebred Spanish Horse Ltd

International Miniature Horse and Pony Society

Appaloosa Horse Club (ApHc) UK Ltd

Shire Horse Society

British Horse Database

British Spotted Pony Society

Exmoor Pony Society

Coloured Horse and Pony Society (UK)

British Percheron Horse Society

British Hanoverian Horse Society

Highland Pony Society

Lipizzaner National Studbook Association of Great Britain

British Equestrian Federation

British Skewbald & Piebald Association

British Miniature Horse Society

Haflinger Society Of Great Britain

Spotted Pony Breed Society (Great Britain)

British Palomino Society

Clydesdale Horse Society

Sport Pony Studbook Society

Icelandic Horse Society Of Great Britain

Suffolk Horse Society

Eriskay Pony Society

United Saddlebred Association UK Ltd

Lusitano Breed Society (Great Britain)

British Morgan Horse Society

Fjord Horse National Stud-book Association of Great Britain

Friesian Horse Association of Great Britain and Ireland Ltd

Oakdales (NI) Ltd

British Harness Racing Club

British Falabella Studbook

British Show Horse Association

British Bavarian Warmblood Association

Caspian Breed Society (UK)

Eriskay Pony (Mother Society)

Comann Each nan Eilean Ltd

Trakehners UK

Gypsy Cob Society

Dales Pony Society

American Quarter Horse Association UK

Spotted Horse and Pony Society

British Association for the Purebred Spanish Horse Ltd

Appaloosa Horse Club (ApHc) UK Ltd

British Spotted Pony Society

British Percheron Horse Society

Lipizzaner National Studbook

Association of Great Britain British Mi
